# Testing Sensitivity of A-Type Residual Current Devices to Earth Fault Currents with Harmonics

**DOI:** 10.3390/s20072044

**Published:** 2020-04-05

**Authors:** Stanislaw Czapp

**Affiliations:** Faculty of Electrical and Control Engineering, Gdańsk University of Technology, 80-233 Gdańsk, Poland; stanislaw.czapp@pg.edu.pl

**Keywords:** current sensor, earth fault, harmonics, low-voltage systems, residual current devices

## Abstract

In many applications, modern current-using equipment utilizes power electronic converters to control the consumed power and to adjust the motor speed. Such equipment is used both in industrial and domestic installations. A characteristic feature of the converters is producing distorted earth fault currents, which contain a wide spectrum of harmonics, including high-order harmonics. Nowadays, protection against electric shock in low-voltage power systems is commonly performed with the use of residual current devices (RCDs). In the presence of harmonics, the RCDs may have a tripping current significantly different from that provided for the nominal sinusoidal waveform. Thus, in some cases, protection against electric shock may not be effective. The aim of this paper is to present the result of a wide-range laboratory test of the sensitivity of A-type RCDs in the presence of harmonics. This test has shown that the behavior of RCDs in the presence of harmonics can be varied, including the cases in which the RCD does not react to the distorted earth fault current, as well as cases in which the sensitivity of the RCD is increased. The properties of the main elements of RCDs, including the current sensor, for high-frequency current components are discussed as well.

## 1. Introduction

**Figure 1:**
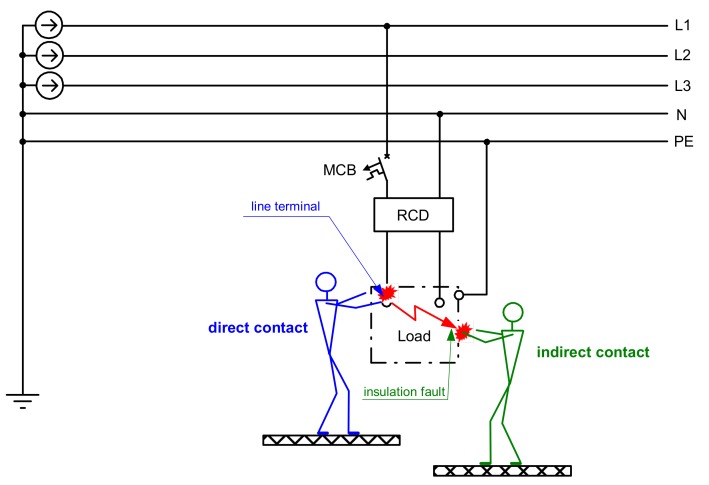
Electric shock due to direct contact with live parts or due to insulation fault (indirect contact); RCD, residual current device; MCB, overcurrent protective device (miniature circuit-breaker).

Electrical and electronic systems from their nature introduce an electric shock hazard. Thus, one of the most important things during the design of such systems is to apply to them effective protection against electric shock. The fundamental rule of electrical safety, both in high- and low-voltage systems, is included in standard EN 61140 [1]: Hazardous-live-parts are not allowed to be accessible, and accessible conductive parts are not allowed to be hazardous-live. For low-voltage systems, detailed rules referring to the protection against electric shock are described in the multipart standard IEC/HD 60364 “Low-voltage electrical installations,” especially in part HD 60364-4-41 [2]. For safety purposes, protection against direct contact (basic protection) and protection against indirect contact (fault protection) have to be used. The most common method of protection against indirect contact is the automatic disconnection of supply. In the case of a line-to-earth fault, dangerous voltage may appear at the metallic enclosure of the current-using equipment, and risk of electrocution, due to indirect contact, exists (Figure 1). To prevent a fatal accident, the power supply has to be disconnected within the time specified in standard HD 60364-4-41 [2] (e.g., in a TN-type low-voltage system, up to 0.4 s for final circuits rated up to 32 A). As a disconnecting device, a residual current device (RCD) may be used. Such a device is the only safety technical measure in the case of direct contact of a live conductor or in the case of touching a line terminal of current-using equipment (load) (Figure 1). Direct contact means that all the current-to-earth flows through the human body. There is no insulation fault; therefore, the body current is too low to activate an overcurrent protective device (miniature circuit-breaker (MCB) in Figure 1) and to switch off the supply. Only a reliable, high-sensitivity RCD may prevent a person from fatal electric shock, provided that the RCD’s rated residual operating current does not exceed 30 mA, which is assumed as the threshold of ventricular fibrillation [3].

To detect an insulation fault, especially a line-to-earth fault, which potentially introduces an electric shock hazard or fire hazard, a suitable protective device of a proper sensitivity level should be selected and applied. The type of protective device and its operational algorithm depends on the type of power network (grounded, ungrounded, overhead line, cable line) [4,5], the necessity of detection of an arc fault [6,7], distorted voltages [8], special signals [9], DC currents [10,11], and the necessity of detection of non-sinusoidal alternating earth fault currents. Special attention should be given to the currents comprising very-low-frequency components [12] or high-order harmonics [13,14,15,16,17,18,19], as in circuits with power electronics converters [20]. If residual current devices are used (they are even obligatory in some circuits), their sensitivity to the non-sinusoidal earth fault (residual) currents has to be verified. The variable-speed drive circuits are the most controversial from the point of view of the selection and operation of RCDs. These circuits generate mixed-frequency earth fault currents [21], the spectrum of which depends on the pulse width modulation (PWM) frequency and actual motor speed. In practical applications, the PWM frequency can be within the range of a few to over 20 kHz. The higher the PWM frequency, the worse the condition for the operation of RCDs. Such currents containing the high-frequency components make tripping characteristics of the most popular RCDs differ from those for the nominal 50/60 Hz [22]. The result of the test of the simplest RCD type presented in [23] shows that their tripping threshold depends on both the RCDs’ magnetic properties of the current sensor and the configuration of the tripping circuit. The increased tripping threshold of RCDs, which can be dangerous for the effectiveness of protection against electric shock, is also noted in the paper [24], and the authors propose a modification of the structure of the RCDs. After modification, proper operation of the analyzed RCD is obtained, but only for a frequency not much higher than the nominal. The problem of the RCD’s operation in the presence of harmonics is indicated in the guide [25]; it strongly underlines that the selection of RCDs to circuits with distorted earth fault current requires a detailed analysis of the earth fault current shapes. The selected RCD has to be able to react to them and, simultaneously, has to be immune to natural earth leakage currents. The manufacturer [25] even recommends special types of RCDs to such circuits. An advanced type of RCD (B-type) is studied in [26], but it is a relatively expensive RCD and used only in specific applications. 

The international standards [27,28,29] recognize the following types of RCDs from the point of view of their sensitivity to the waveform shape:

- AC-type: Intended for 50/60 Hz sinusoidal residual currents only,
- A-type: Intended for waveforms, the same as for AC-type RCDs, and for DC-pulsating residual currents with a smooth DC component up to 6 mA,
- F-type: Intended for waveforms, the same as for A-type RCDs (but smooth DC component can be increased, up to 10 mA) and mixed-frequency residual currents; the RCD should be supplied from a single-phase.
- B-type: Intended for residual currents: Sinusoidal up to 1000 Hz, mixed-frequency, pulsating DC, as well as smooth DC.

It can be assumed that A-type RCDs are now the most popular RCDs utilized in low-voltage systems. At least such a type of RCD has to be used in some countries, e.g., in Germany and Denmark [30,31]. The A-type RCDs are also installed in circuits with power electronic converters, but, from the author’s experience, in some cases, such RCDs may not give effective protection against electric shock.

The aim of this paper is to present and comment on the results of the wide laboratory test of the A-type RCD’s sensitivity to the non-sinusoidal earth fault (residual) currents. The laboratory test shows that the sensitivity of the A-type RCDs depends on the order and content of the harmonic, and their real tripping current may significantly exceed the normative threshold provided for the 50 Hz sinusoidal waveform. The negative impact of harmonics on the RCDs’ current sensor and the tripping relay is discussed. Moreover, unexpected positive responses of the RCDs to the strongly distorted special residual current waveforms are presented. Detailed analysis of the operation of RCDs under non-sinusoidal current contributes to a more accurate evaluation of the effectiveness of protection against electric shock in modern low-voltage electrical circuits, as well as indicates a direction in the necessary modification of the structure of the RCDs in order to achieve better tripping parameters in the presence of harmonics. 

## 2. The Scope of the Investigation and Its Results

### 2.1. General Information

**Figure 2:**
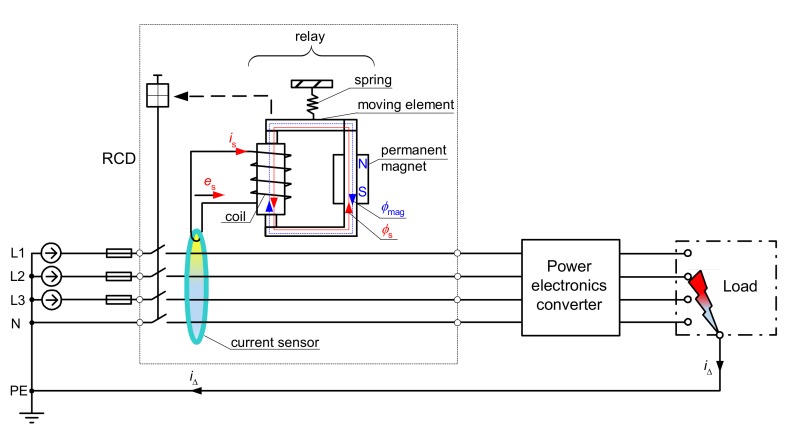
Residual current device (RCD) protecting a 3-phase circuit; *e*_s_—induced voltage in the secondary winding of the current sensor, *i*_s_—secondary current flowing through the relay, *φ*_mag_—magnetic flux from the permanent magnet, *φ*_s_—magnetic flux from the secondary current, *i*_Δ_—residual current.

A residual current device should be connected to the live conductors (line L and neutral N), as it is presented in Figure 2. The protective conductor PE has to go outside the RCD. In the case of an earth fault in the protected circuit, the earth fault current *i*_Δ_ (residual current for the RCD) is a source of an unbalanced current state in the current sensor (summation current transformer) and produces magnetic flux in its iron core. The magnetic flux produces secondary voltage *e*_s_, which is a source of secondary current *i*_s_ flowing through the relay. The relay mainly comprises a permanent magnet, a coil, a spring, and a moving element. The most common utilized RCDs have a rated residual operating current of *I*_Δn_ = 30 mA, and they are voltage-independent. This means that the power necessary for operation of the RCDs is derived only from the residual current. In the case of 30 mA RCDs, the power delivered to the relay is extremely low, so it forces the utilization of the relays, especially with the permanent magnet.

When there is no residual current in the protected circuit, the permanent magnet producing the magnetic flux *φ*_mag_ keeps the moving element of the relay in the closed position. While a residual current flows, the secondary current *i*_s_ produces magnetic flux *φ*_s_ that is superimposed on the flux *φ*_mag_ (see respective lines in the relay in Figure 2). If the magnetic flux is sinusoidal, in the one half-wave, it has the same direction as the constant-value magnetic flux from the permanent magnet. In turn, in the second half-wave, the magnetic flux *φ*_s_ has the opposite direction and reduces the flux *φ*_mag_. If this reduction is significant, the spring pulls away from the moving element of the relay, and by consequence, the main circuit becomes open.

In circuits with distorted residual currents, the tripping threshold of the RCDs changes. The next section presents a theoretical analysis of the RCDs’ relay operation when the residual current, as well as the secondary current, comprises the following components:

- fundamental + 3^rd^ harmonic (content 50%); phase angle of the 3^rd^ harmonic: 0°, 45°, 90°, 180°;
- fundamental + 11^th^ harmonic (content 50%); phase angle of the 11^th^ harmonic: 0°, 45°, 90°, 180°;
- fundamental + 49^th^ harmonic (content 50%); phase angle of the 49^th^ harmonic: 0°, 180°.

After this, the results of the laboratory test are presented. The tripping threshold of a group of A-type RCDs has been evaluated, for the following testing currents:

- fundamental + 3^rd^ harmonic,
- fundamental + 5^th^ harmonic,
- fundamental + 7^th^ harmonic,
- fundamental + 11^th^ harmonic,
- fundamental + 23^rd^ harmonic,
- fundamental + 49^th^ harmonic.

The above-mentioned harmonics were selected (as an example) to test based on the study of the literature, as well as from the practical experience of the author. Relatively high-frequency harmonics (here, 23^rd^ and 49^th^) reflect the components produced by a power converter modulation (PWM frequency, its multiple and interharmonics). 

During this test, the content of the harmonic (with reference to the fundamental) was consecutively forced: 50%, 100%, and 200%, and the phase angle of the harmonic was consecutively set to 0°, 45°, 90°, 135°, 180°, 225°, 270°, and 315°.

The tripping threshold of the two selected RCDs has also been verified for testing waveforms comprising many harmonics (as in a circuit with variable-speed drives). Furthermore, for comparison of the behavior of the RCDs, their tripping threshold under a special distorted waveform—bidirectional waveform characterized by various trigger delay angles—is presented as well. 

Finally, the main sources of the changes in the tripping threshold of RCDs in circuits with distorted residual currents are presented and discussed.

### 2.2. Theoretical Analysis of the RCDs’ Relay Operation in the Presence of Harmonics

Distorted residual current can be described by the following dependency:

*i*_Δ_ =⋅*I*_Δamp_ ⋅[sin(*ωt* + *ψ*_1_) + *C*_3_ ⋅ sin(3*ωt* + *ψ*_3_) + *C*_5_ ⋅ sin(5*ωt* + *ψ*_5_) + … + *C*_h_ ⋅ sin(*hωt* + *ψ*_h_)] (1)

where:

*i*_Δ_ : Residual current as a function of time,

*I*_Δ amp_ : Amplitude of the residual current,

*C_3_*, *C*_5_, … *C*_h_ : The content of the amplitude-to-fundamental for the 3rd, 5th, …, *h*-th harmonic

*ψ*_3_, *ψ*_5_, … *ψ*_h_ : Phase angle of the 3rd, 5th, …, *h*-th harmonic.

**Figure 3:**
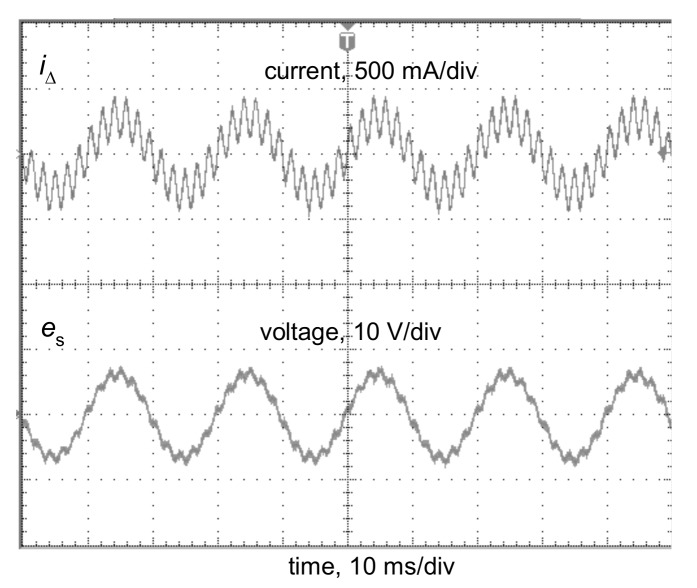
Oscillogram of the induced secondary voltage *e*_s_ in the secondary winding of the current sensor from a 300 mA A-type RCD for the residual current *i*_Δ_ composed of the fundamental (50 Hz) and 50% content of the 11^th^ harmonic.

However, the operation of the relay of the RCD (Figure 2) depends on the secondary current *i*_s_, which flows at the secondary side of the current sensor. To verify the transformation quality of the iron core of the A-type current sensor, a laboratory test of the induced secondary voltage *e*_s_, generated by the distorted residual current *i*_Δ_, has been performed. It may be concluded that the shape of the induced voltage *e*_s_ is relatively divergent with the shape of the residual current *i*_Δ_; Figure 3 presents, as an example, a selected result of this test when the residual current *i*_Δ_ is composed of the fundamental (50 Hz) and the 50% content of the 11^th^ harmonic. In further analysis, it is assumed that the secondary current *i*_s_ has a similar shape as the residual current *i*_Δ_. 

The secondary current *i*_s_ flowing in a coil, which is a part of the RCD’s relay, produces magnetic flux *φ*_s_:

(2) $$\varphi_{s}=\frac{i_{s}\cdot n_{c}}{R_{m}},$$

where:

*i*_s_ : Current, which flows at the secondary side of the current sensor,

*n*_c_ : Number of turns in the relay coil,

*R*_m_ : The reluctance of the relay magnetic path.

Further, the magnetic force *F*_s_ generated by the magnetic flux *φ*_s_ in the relay can be described by the following expression [32]:

(3) $$F_{s}=\frac{{\left(\varphi_{s}\right)}^{2}}{s_{\mathrm{core}}\cdot \mu_{0}},$$

where:

*φ*_s_ : Magnetic flux generated by the secondary current *i*_s_,

*s*_core_ : Cross-sectional area of the relay core,

*μ*_0_ : Magnetic constant (permeability of vacuum),

but the magnetic force *F*_mag_ from the magnetic flux *φ*_mag_ produced by the permanent magnet is represented by the following formula:

(4) $$F_{\mathrm{mag}}=\frac{{\left(\varphi_{\mathrm{mag}}\right)}^{2}}{s_{\mathrm{core}}\cdot \mu_{0}},$$

where:

*φ*_mag_ : Magnetic flux generated by the permanent magnet,

*s*_core_ : Cross-sectional area of the relay core, 

*μ*_0_ : Magnetic constant (permeability of vacuum).

Assuming that the spring pulls the moving element of the relay with the force *F*_spring_, the resultant force *F*_res_ from the interaction, permanent magnet–spring–secondary current, is as follows:

(5) $$F_{\mathrm{res}}=\left[F_{\mathrm{mag}}-F_{\mathrm{spring}}\right]-F_{s}=\left[\frac{{\left(\varphi_{\mathrm{mag}}\right)}^{2}}{s_{\mathrm{core}}\cdot \mu_{0}}-F_{\mathrm{spring}}\right]-\frac{{\left(\varphi_{s}\right)}^{2}}{s_{\mathrm{core}}\cdot \mu_{0}}=\frac{{\left(\varphi_{\mathrm{mag}}\right)}^{2}-{\left(\varphi_{s}\right)}^{2}}{s_{\mathrm{core}}\cdot \mu_{0}}-F_{\mathrm{spring}}.$$

**Figure 4:**
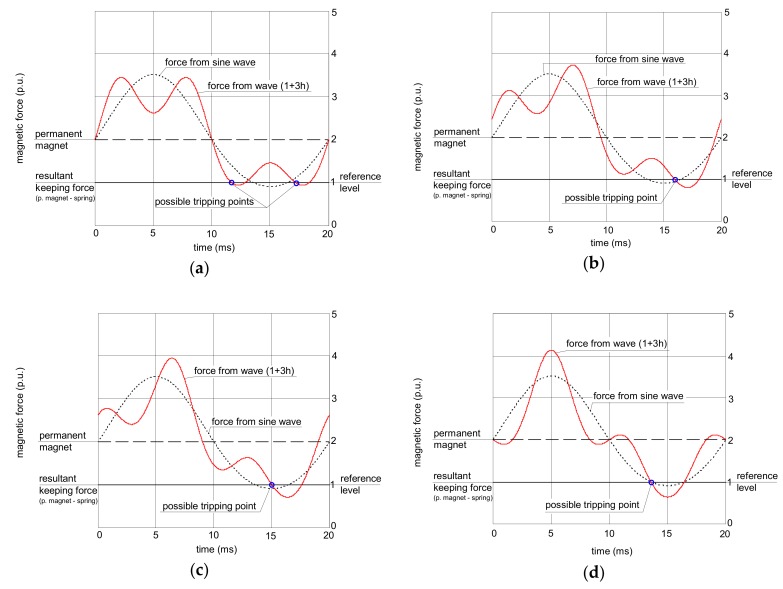
Interaction between forces in the electromechanical relay of the RCD for earth fault current waveforms comprising the fundamental and 50% of the 3^rd^ harmonic. Phase angle of the 3^rd^ harmonic: (**a**) 0°; (**b**) 45°; (**c**) 90°; (**d**) 180°.

**Figure 5:**
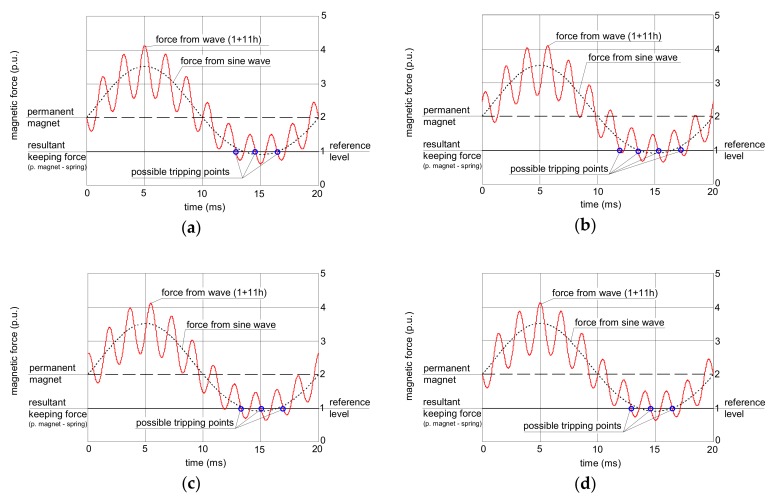
Interaction between forces in the electromechanical relay of the RCD for earth fault current waveforms comprising the fundamental and 50% of the 11^th^ harmonic. The phase angle of the 11^th^ harmonic: (**a**) 0°; (**b**) 45°; (**c**) 90°; (**d**) 180°.

**Figure 6:**
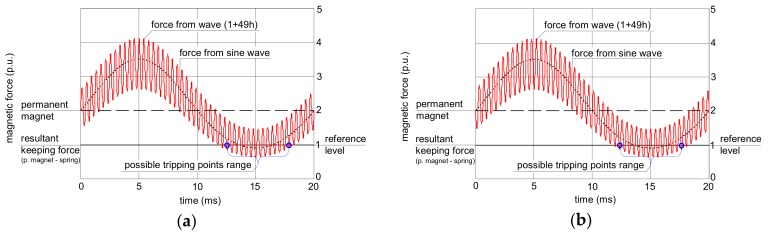
Interaction between forces in the electromechanical relay of the RCD for earth fault current waveforms comprising the fundamental and 50% of the 49^th^ harmonic. The phase angle of the 49^th^ harmonic: (**a**) 0°; (**b**) 180°.

Theoretical analysis of the operation of the RCDs’ relay is conducted graphically (Figure 4, Figure 5 and Figure 6). In every figure, a reference magnetic force from the sinusoidal current (dotted line) is included, and on its background, the force produced by a non-sinusoidal current is presented. All forces are scaled in relative units. 

Figure 4 presents an analysis of the relay operation in the case of the waveform comprising the fundamental and the 3^rd^ harmonic. In all the analyses, the content of the higher harmonics is 50% (percentage-to-fundamental; the fundamental has 50 Hz). If the subfigures (a), (b), (c), and (d) from Figure 4 are compared, one can see that the peak value of the force generated by the non-sinusoidal current depends on the phase angle of the higher harmonics. 

In Figure 4a, the force from the wave 1+3h with phase angle 0° crosses the resultant keeping force from the permanent magnet and the spring but only with a slight excess. In the case of Figure 4d (the wave 1+3h with phase angle 180°), this excess is the highest. The tripping threshold of the relay depends on the peak value of the secondary current; therefore, for the waveform from Figure 4a, the tripping current of the RCD is expected to be higher (worse sensitivity) than for the waveform from Figure 4d.

The analyzed waveform comprising the fundamental and the 11^th^ harmonic gives different conclusions (Figure 5). The effect of the higher harmonic phase angle is not as significant as in the case of the 3^rd^ harmonic; the peak value of the force produced by the non-sinusoidal current is almost the same in every case from Figure 5.

Even less influence of the phase angle of the higher harmonics is observed for the 49^th^ harmonic (Figure 6). The shapes of the force waveform are practically the same regardless of the value of the phase angle of the 49^th^ harmonic; it should give the same level of tripping current for all the phase angles.

### 2.3. Laboratory Verification of the Tripping Current in the Presence of Harmonics

**Figure 7:**
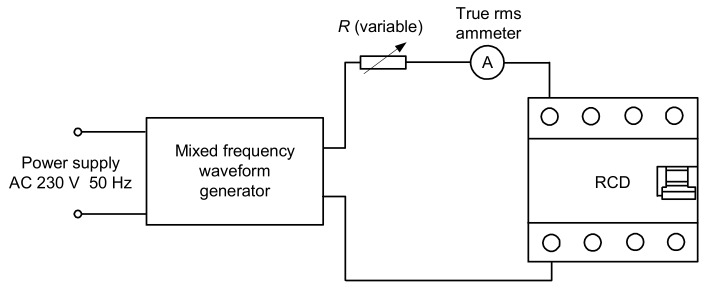
Diagram of the laboratory stand for RCD testing.

The sensitivity of the A-type RCDs (*I*_Δn_ = 30, 100, and 300 mA) has been tested with the use of the laboratory stand, the general structure of which is presented in Figure 7. This paper includes the results of the test for the eight RCDs, which are representative and the most interesting.

**Figure 8:**
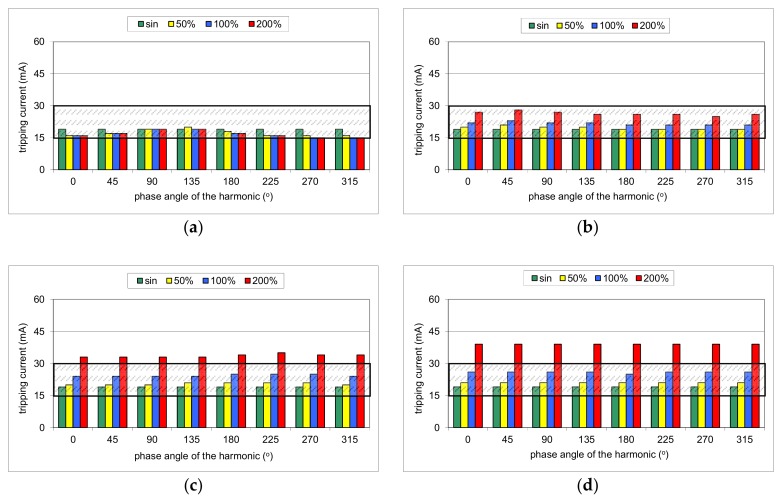

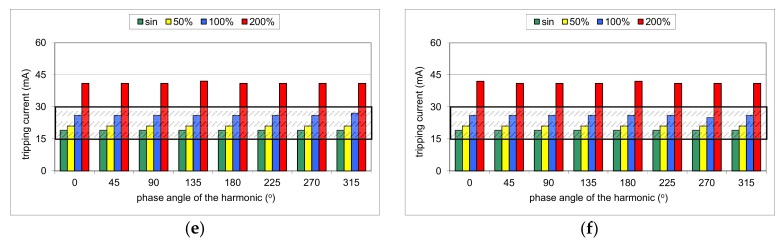
Tripping current of the A-type 30 mA RCD (no. RCD30_1) for testing waveform comprising the fundamental and the following high-frequency components: (**a**) 3^rd^ harmonic; (**b**) 5^th^ harmonic; (**c**) 7^th^ harmonic; (**d**) 11^th^ harmonic; (**e**) 23^rd^ harmonic; (**f**) 49^th^ harmonic. Tested percentages of harmonic-to-fundamental: 50%, 100%, and 200%, and phase angles of the harmonic: 0°, 45°, 90°, 135°, 180°, 225°, 270°, 315°.

**Figure 9:**
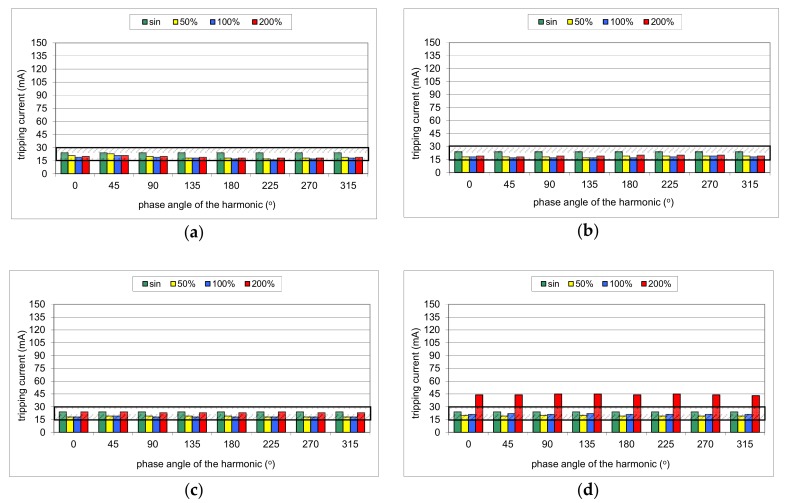

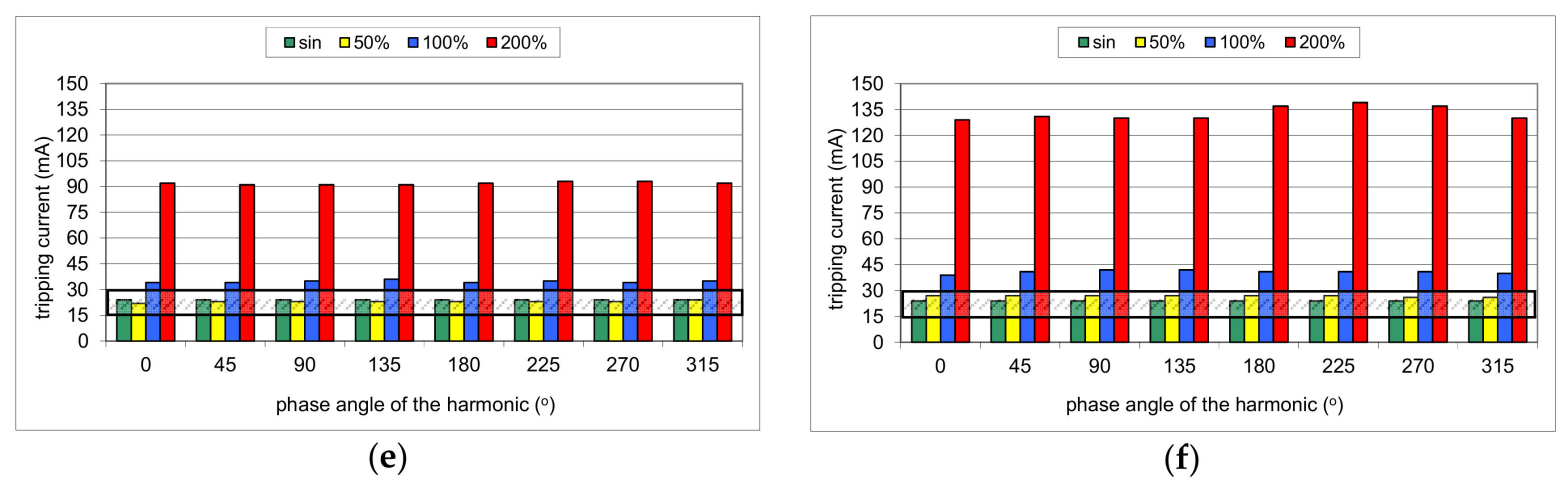
Tripping current of the A-type 30 mA RCD (no. RCD30_2) for testing waveform comprising the fundamental and the following high-frequency components: (**a**) 3^rd^ harmonic; (**b**) 5^th^ harmonic; (**c**) 7^th^ harmonic; (**d**) 11^th^ harmonic; (**e**) 23^rd^ harmonic; (**f**) 49^th^ harmonic. Tested percentages of harmonic-to-fundamental: 50%, 100%, and 200%, and phase angles of the harmonic: 0°, 45°, 90°, 135°, 180°, 225°, 270°, 315°.

**Figure 10:**
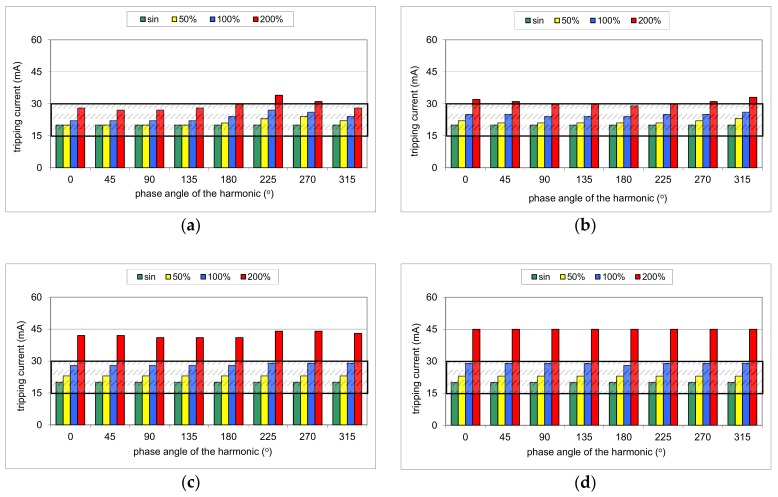

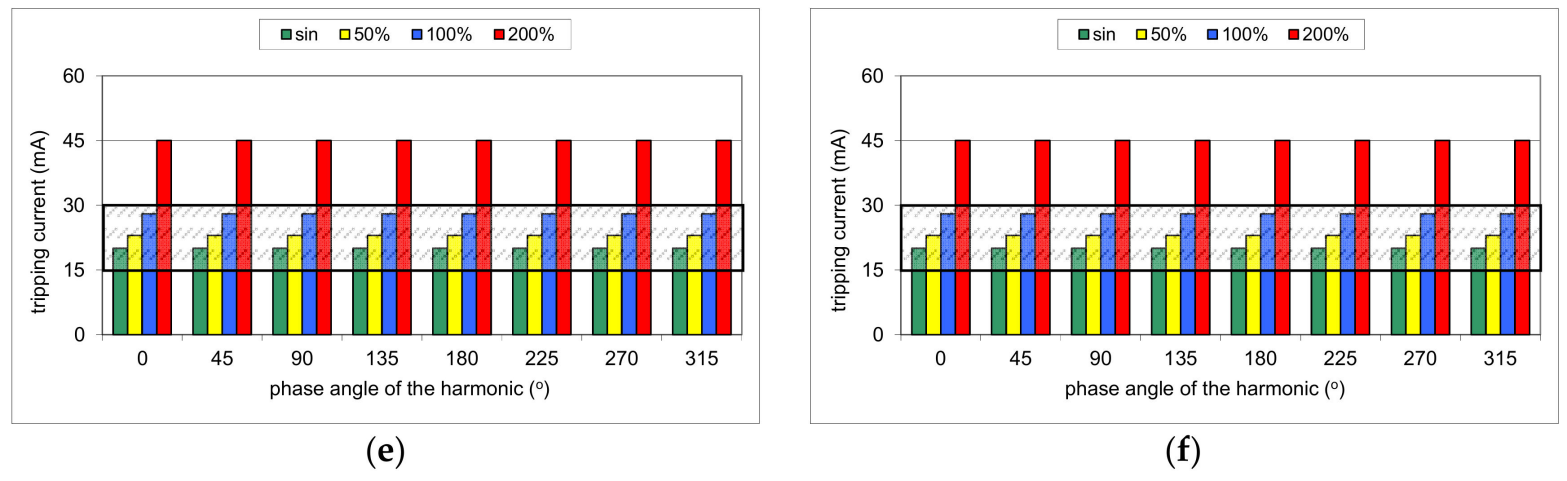
Tripping current of the A-type 30 mA RCD (no. RCD30_3) for testing waveform comprising the fundamental and the following high-frequency components: (**a**) 3^rd^ harmonic; (**b**) 5^th^ harmonic; (**c**) 7^th^ harmonic; (**d**) 11^th^ harmonic; (**e**) 23^rd^ harmonic; (**f**) 49^th^ harmonic. Tested percentages of harmonic-to-fundamental: 50%, 100%, and 200%, and phase angles of the harmonic: 0°, 45°, 90°, 135°, 180°, 225°, 270°, 315°.

**Figure 11:**
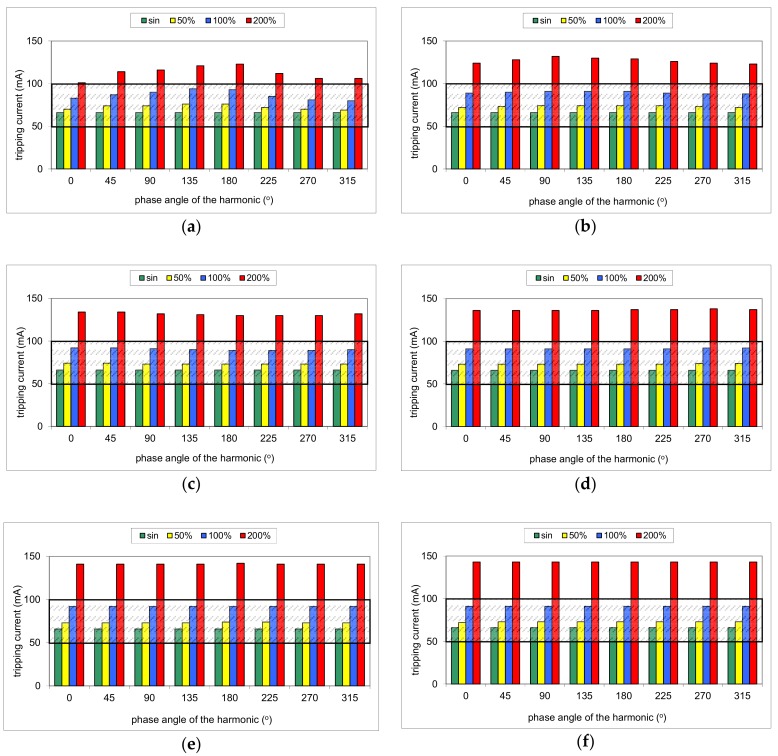
Tripping current of the A-type 100 mA RCD (no. RCD100) for testing waveform comprising the fundamental and the following high-frequency components: (**a**) 3^rd^ harmonic; (**b**) 5^th^ harmonic; (**c**) 7^th^ harmonic; (**d**) 11^th^ harmonic; (**e**) 23^rd^ harmonic; (**f**) 49^th^ harmonic. Tested percentages of harmonic-to-fundamental: 50%, 100%, and 200%, and phase angles of the harmonic: 0°, 45°, 90°, 135°, 180°, 225°, 270°, 315°.

**Figure 12:**
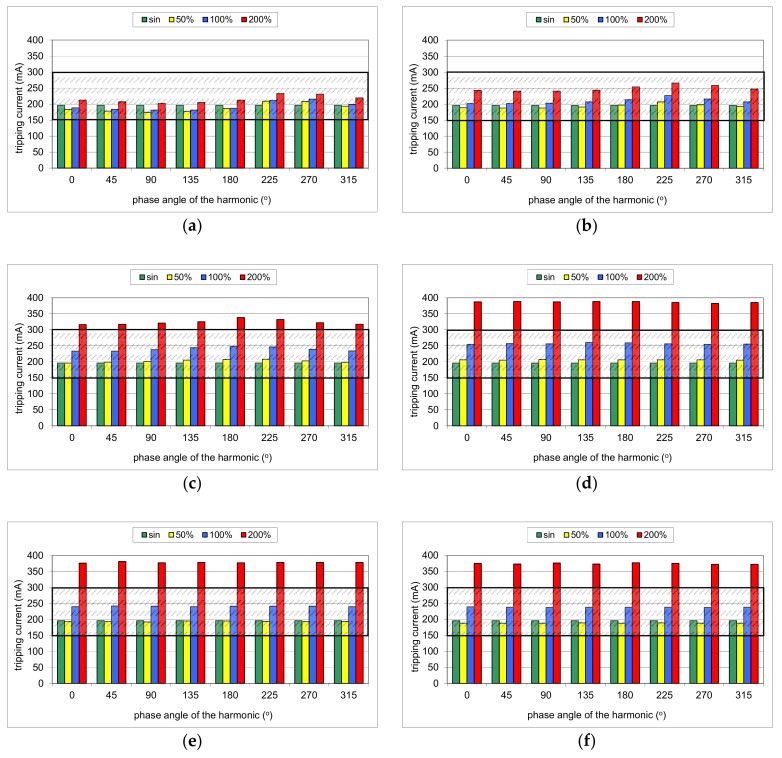
Tripping current of the A-type 300 mA RCD (no. RCD300_1) for testing waveform comprising the fundamental and the following high-frequency components: (**a**) 3^rd^ harmonic; (**b**) 5^th^ harmonic; (**c**) 7^th^ harmonic; (**d**) 11^th^ harmonic; (**e**) 23^rd^ harmonic; (**f**) 49^th^ harmonic. Tested percentages of harmonic-to-fundamental: 50%, 100%, and 200%, and phase angles of the harmonic: 0°, 45°, 90°, 135°, 180°, 225°, 270°, 315°.

During the first part of the test, the mixed-frequency waveform generator has been utilized for the production of the fundamental waveform and one of the following harmonics: 3^rd^, 5^th^, 7^th^, 11^th^, 23^rd^, and 49^th^. The content of the particular harmonic was consecutively set to 50%, 100%, and 200% with reference to the fundamental. The phase angle of the harmonic was set within the range 0°–315° with a 45° step. The mixed-frequency current was slowly increased with the use of the resistor *R*. As a reference value, the tripping current of each RCD was checked for the 50 Hz sinusoidal waveform. According to the standard [28], the tripping current for the sinusoidal waveform should be within the range (0.5–1.0)*I*_Δn_, i.e., 15–30 mA for an RCD of *I*_Δn_ = 30 mA. Figure 8, Figure 9, Figure 10, Figure 11 and Figure 12 present the results of this part of the test for selected RCDs, and the aforementioned range is marked as a shaded area.

The result of the tests of RCD30_1 (Figure 8) shows that its tripping threshold for the testing waveform containing the 3^rd^ harmonic depends on the phase angle of this harmonic, which confirms the above-conducted theoretical analysis. In any of the trials, the tripping threshold is within the normative range as for the sinusoidal waveform (0.5–1.0)*I*_Δn_ (Figure 8a). For the phase angle of the 3^rd^ harmonic equal to 270° or 315°, the RCD tripped almost at the lower permissible limit 0.5*I*_Δn_ when the content of the harmonic was 100% or 200%. In these cases, it is a kind of increase in RCD sensitivity.

The effect of the phase angle of the higher harmonic on the tripping current is also noticeable in the case of the 5^th^ harmonic (Figure 8b). However, the content of the harmonic equal to 100% or 200% makes that tripping threshold rise in comparison to a pure sinusoidal waveform. For a content of 200%, it is close to the upper limit 1.0*I*_Δn_. For the other harmonics (7^th^, Figure 8c; 11^th^, Figure 8d; 23^rd^, Figure 8e; 49^th^, Figure 8f, the tripping current for a particular harmonic does not depend on the phase angle of the harmonic, which confirms the theoretical analysis as well. However, for a high content of the harmonic (200%), RCD30_1 tripped at a level higher than the upper limit (30 mA). It signifies that in the adverse condition (high content of higher-order harmonics), such an RCD may not give sufficient protection against electric shock. 

The problem of the strongly increased tripping threshold is clearly seen in the case of RCD30_2 (Figure 9). As far as for harmonics 3^rd^ (Figure 9a), 5^th^ (Figure 9b), and 7^th^ (Figure 9c), this RCD reacts very well (within the range of 0.5–1.0*I*_Δn_), but for the other harmonics, especially for a content of 200% of the 23^rd^ or the 49^th^ harmonic, the tripping current rises rapidly. For the 49^th^ harmonic, it is around 6–7 times higher than for the pure sine waveform.

More even tripping characteristics are observed for RCD30_3 (Figure 10), especially for harmonics 11^th^, 23^rd^, and 49^th^. These three groups of results (Figure 10d–f) are almost the same. Furthermore, for the highest content (200%) of the aforementioned harmonics (11^th^, 23^rd^, and 49^th^), the tripping threshold is stable (around 45 mA). Unfortunately, what is unexpected is that the content of 200% of the 3^rd^ harmonic (relatively low-order harmonic) may give a tripping current exceeding 30 mA (upper limit for the 50 Hz sine wave) (Figure 10a).

Similar tripping behavior is noticed in the case of RCD100 (Figure 11). However, an unfavorable effect of the harmonics is recorded even for the relatively low-order harmonics. In the case of the 3^rd^ harmonic and its content equal to 200%, the tripping threshold is higher than *I*_Δn_ = 100 mA for every tested phase angle.

The test of RCD300_1 (Figure 12) has given typical expected results; relatively low-order harmonics (here 3^rd^ and 5^th^) superimposed on the fundamental give a real tripping current dependent on the phase angle of the harmonic, but it is kept within the range (0.5–1.0)*I*_Δn_. High-order harmonics (11^th^, 23^rd^, and 49^th^), with their content very high, result in a tripping current that exceeds the upper limit 1.0*I*_Δn_. 

**Figure 13:**
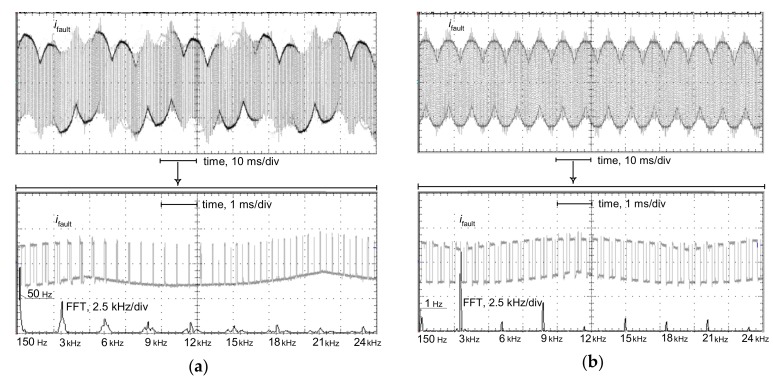
Oscillograms of the line-to-earth fault current waveform *i*_fault_ and its FFT analysis in the case of an earth fault at the output terminals of the frequency converter supplying a motor: (**a**) operating frequency of 50 Hz (nominal motor speed); (**b**) operating frequency of 1 Hz (very low motor speed); pulse width modulation (PWM) frequency of 3 kHz; line-to-earth connection via resistance of 1 kΩ (conventional human body resistance).

The tripping current has also been tested for the testing waveform comprising many harmonics. Figure 13 presents oscillograms of the earth fault current at the terminal of the motor supplied via a frequency converter (variable-speed drive circuit). The earth fault was carried out through the resistance 1 kΩ—it reflects direct contact of a human with the live conductor. The PWM frequency is equal to 3 kHz. The earth current is built from low-order harmonics, as well as very-high-order harmonics resulting from the PWM frequency. Note that this current also contains a multiple of the PWM frequency, even above 20 kHz here. Moreover, the spectrum of the waveform depends on the actual motor speed (nominal motor speed, Figure 13a; very low motor speed, Figure 13b). The highest content of high-order harmonics occurs for very low motor speed (1 Hz). In the test of the operation of the RCDs, their tripping current has been verified for the earth current waveforms (reflected in the laboratory generator) occurring in the case of the nominal motor speed (50 Hz), decreased motor speed (25 Hz), and strongly decreased motor speed (1 Hz). The PWM frequency was adopted to be equal to 1 kHz.

**Figure 14:**
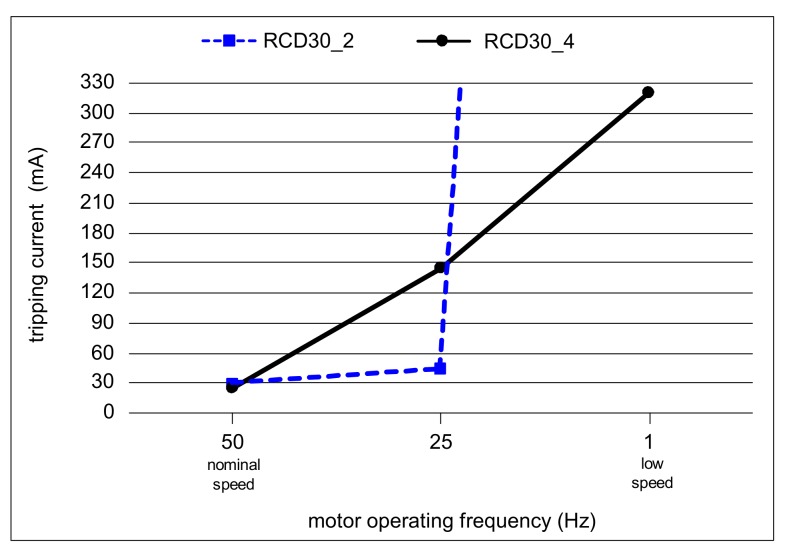
Tripping current of two RCDs (*I*_Δn_ = 30 mA) for testing currents comprising many harmonics as in a variable-speed drive circuit.

Results of the test of two 30 mA RCDs, for the multiple-harmonics testing current, are presented in Figure 14. For the earth current waveform occurring during nominal motor speed (50 Hz), both tested RCDs have a tripping current not exceeding 30 mA. In the case of the supplying frequency reflecting decreased motor speed (25 Hz), the tripping current of RCD30_2 is 44 mA, whereas that of RCD30_4 is as much as 145 A. Very unfavorable behavior of RCD30_2 was observed in the case of the testing waveform reflecting the strongly decreased motor speed (1 Hz). The maximal forced testing current was over 3 A (one hundred times higher than the rated residual operating current of the tested RCDs), but RCD30_2 did not operate. Fortunately, RCD30_4 tripped, but at 320 mA. This test has shown that the behavior of A-type RCDs in the presence of harmonics can be unexpected. Current harmonics, especially high-order harmonics, increase the tripping threshold of the RCDs; in some cases, the RCDs may not react to the earth fault currents having a relatively high content of high-order harmonics. Additionally, some RCDs may have increased tripping current even in the presence of relatively low-order harmonics (3^rd^, 5^th^, or 7^th^).

### 2.4. Discussion: Sources of the Varied Tripping Current in the Presence of Harmonics

**Figure 15:**
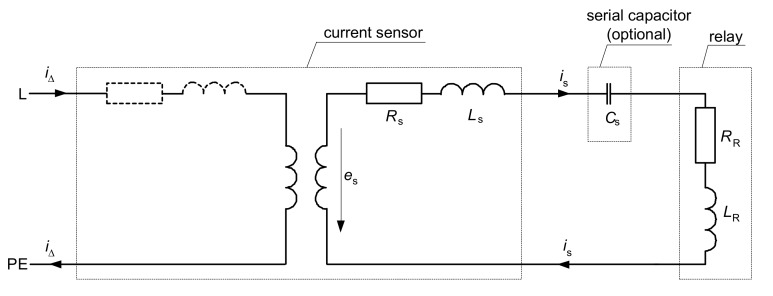
Equivalent circuit of the RCD; *i*_Δ_—residual (primary) current, *i*_s_—secondary current, *e*_s_— voltage induced in the secondary winding of the current sensor, *R*_s_—resistance of the secondary winding, *L*_s_—inductance of the secondary winding, *R*_R_—resistance of the relay, *L*_R_—inductance of the relay, *C*_s_—serial capacitor (optional).

Analysis of the effect of harmonics on the main elements of the RCDs is conducted based on the equivalent circuit presented in Figure 15. In the analysis, the most important are as follows:

- magnetic properties of the current sensor (transformer) for various frequencies,
- the configuration of the secondary circuit: Whether only the relay is installed or additional electronic elements (capacitors serial/parallel) are installed as well,
- parameters of the relay, especially for various frequencies.

**Figure 16:**
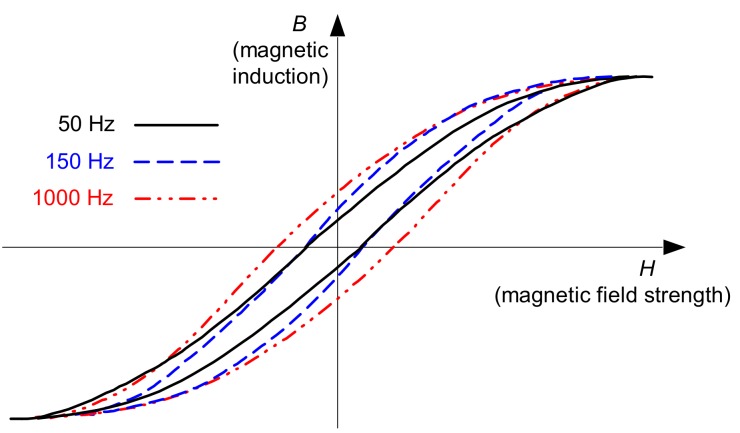
Measured hysteresis loops of the current sensor of the 30 mA A-type RCD, for frequencies of 50, 150, and 1000 Hz.

To verify the magnetic properties of the current sensor of the RCDs, hysteresis loops of the selected A-type current sensor (from the RCD of *I*_Δn_ = 30 mA) have been measured for the following frequencies: 50, 150, and 1000 Hz. Results of the measurements are presented in Figure 16. 

One can see that for higher frequencies, the hysteresis loop shapes are different than for the fundamental frequency of 50 Hz. The most important is the fact that for the relatively high frequency (1000 Hz), the shape is wider compared to 50 Hz. Thus, to achieve sufficient magnetic induction for such a high frequency, magnetic field strength has to be higher than for the nominal/fundamental frequency. It can be one of the sources of the significantly higher tripping current of the tested RCDs when the testing waveform contains the 23^rd^ or 49^th^ harmonic (see Figure 8, Figure 9, Figure 10, Figure 11 and Figure 12). 

**Figure 17:**
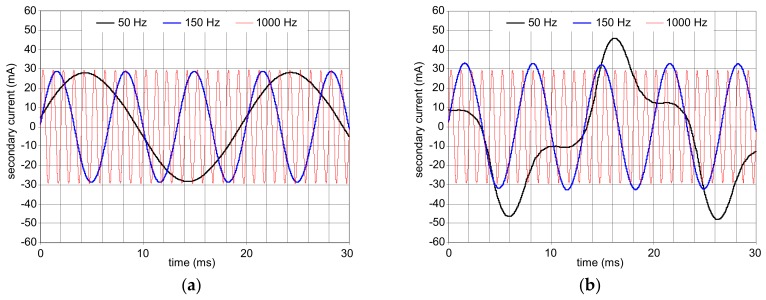
Results of the computer simulations of the secondary current (*i*_s_ in Figure 2) flowing through the electromechanical relay: (**a**) No serial capacitor in the secondary circuit; (**b**) serial capacitor in secondary circuit is applied; values of the residual current frequency: 50, 150, and 1000 Hz [33].

Additional matching elements (as capacitors) in the secondary circuit of the RCD may also influence the RCDs tripping negatively in the presence of high-frequency components. Figure 17 presents results of the previous investigations [33], in which the secondary current waveform was simulated in the circuit without a serial matching capacitor (Figure 17a) and with this capacitor (Figure 17b). This capacitor gives the possibility for delivering the increased power (increased secondary current, especially the peak value, which is the most important) to the relay. This improves the sensitivity of the RCDs. Increased secondary current, in comparison to the case without the capacitor, has been obtained, but only for the fundamental frequency 50 Hz (compare black traces: Figure 17a versus Figure 17b). The favorable effect of the capacitor in the secondary circuit of the RCD is practically not observed for the other tested frequencies: 150 and 1000 Hz. The corresponding current waveforms are almost identical. 

The secondary current value also depends on the induced secondary voltage *e*_s_ and the impedance of the relay, as a function of the frequency. Induced secondary voltage *e*_s_ in the secondary winding of the current sensor can be described by the following equation:

*e*_s_ = 4.44 *n*_s_*f φ*_M_, (6)

where:

*n*_s_ : Number of turns in the secondary winding of the current sensor,

*f* : Frequency of the current waveform,

*φ*_M_ : The magnetic flux in the core of the current sensor, coupled with the primary winding and the secondary winding of the current sensor.

**Figure 18:**
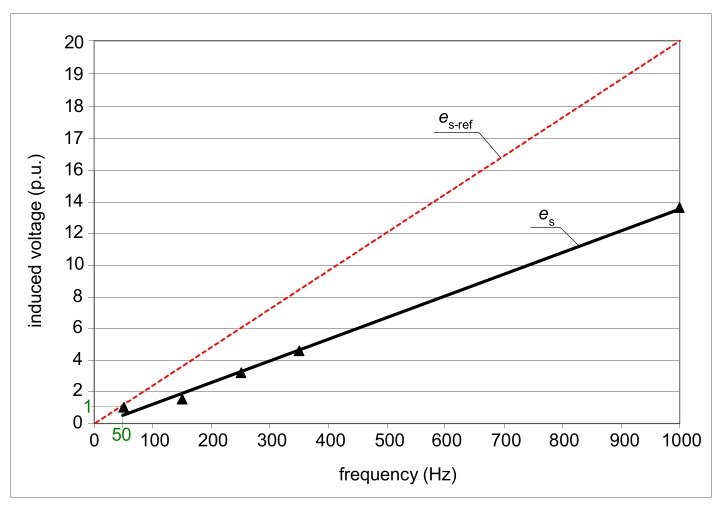
Variation (measured) in the induced secondary voltage *e*_s_ of the A-type current sensor for the frequency range of the residual current *f* = 50–1000 Hz; *e*_s-ref_ (dotted red line), reference line for an ideal current sensor (transformer).

**Figure 19:**
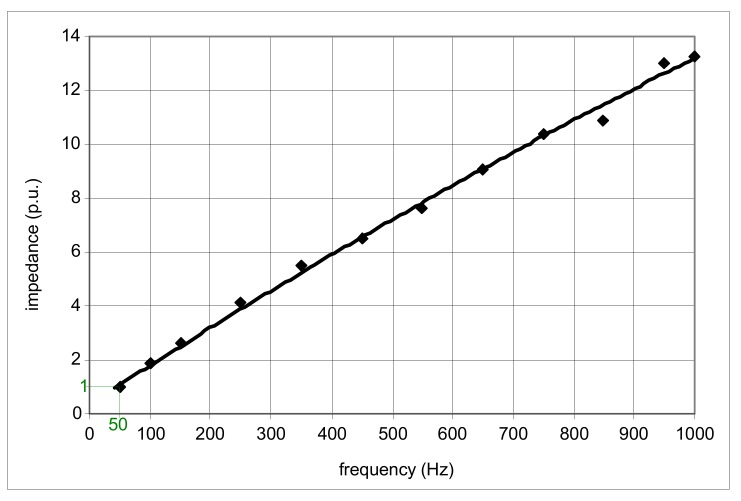
Impedance variation (measured) of the example relay (just before tripping) of the 30 mA A-type RCD, within the frequency range *f* = 50–1000 Hz.

Theoretically (ideally), the induced voltage *e*_s_ should vary in the same way as the frequency *f* varies. In practice, this dependence is different. Figure 18 presents results of the measured induced voltage (relative, with reference to those for the frequency of 50 Hz) for the A-type current sensor. One can see that the variation in frequency from 50 to 1000 Hz (20×) gives an increase in the induced voltage of only around 14 times. This phenomenon, as well as increasing impedance of the relay as a function of frequency (Figure 19), may result in the increased tripping current of A-type RCDs for the earth fault currents with harmonics, especially high-order harmonics.

## 3. Behavior of RCDs under Residual Currents with Controlled Delay Angle

**Figure 20:**
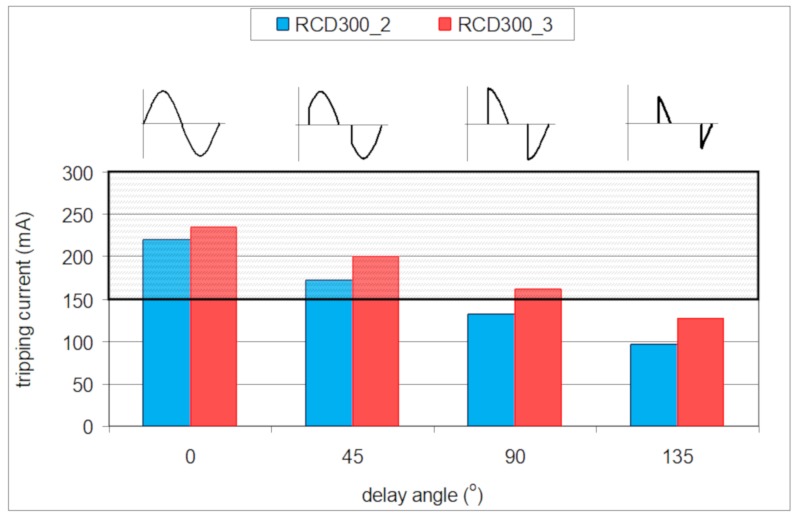
Tripping current of two 300 mA A-type RCDs for the following current delay angles: 0°, 45°, 90°, and 135°.

Power control of current-using equipment may be realized with the use of electronic converters, which control the delay angle of the load current. This way of power control is used, among others, in lighting circuits. The higher the value of the current delay angle, the more distorted the current. Taking into account the above considerations, related to the behavior of RCDs in the presence of harmonics, the first conclusion may appear that strongly distorted residual current, having a high delay angle value, would also give deteriorated sensitivity of the RCD, even above the upper limit 1.0*I*_Δn_. To verify this assumption, a laboratory test of two 300 mA A-type RCDs was conducted for the following current delay angles of the residual current: 0°, 45°, 90°, and 135°, and its results are presented in Figure 20.

For a sinusoidal testing current (current delay angle equal to 0°), the tripping current of both the tested RCDs is almost in the middle of the normative characteristic (0.5–1.0)*I*_Δn_. However, the higher the value of the current delay angle, the lower the tripping current. What is interesting is that for a current delay angle equal to 90°, the tripping current of the RCD300_2 is below the lower limit 0.5*I*_Δn_ assigned to the sinusoidal waveform. In the case of the angle 135°, both tested RCDs have a tripping current below this limit. Thus, strongly distorted symmetrical residual current, having a high value of the delay angle, is very easily detected by the A-type RCDs. The effective protection against electric shock is not compromised; only unexpected nuisance tripping of the RCDs, for high values of the delay angle, may occur. 

**Figure 21:**
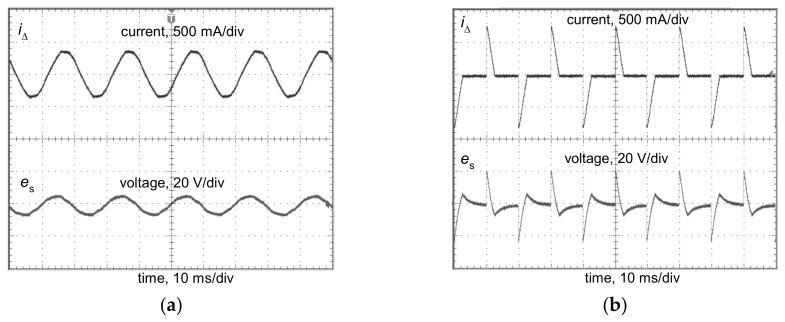
Oscillograms of the induced secondary voltage *e*_s_ in the secondary winding of the current sensor from a 300 mA A-type RCD, for the following current delay angles of the residual current: (**a**) 0°; (**b**) 135°; in both cases, the rms values of the residual current *i*_Δ_ are the same.

Tripping current of the tested RCDs, under the lower limit of 0.5*I*_Δn_, is associated with the relation of the peak value of the current waveform to its rms value. One must remember that the tripping threshold of the RCDs depends on the peak value of the residual current. If the residual current has increased current delay angle, the ratio peak-to-rms is higher than for a sinusoidal waveform. Figure 21 presents oscillograms of the induced secondary voltage *e*_s_ in the secondary winding of the current sensor dismounted from a 300 mA A-type RCD. In both cases (delay angle 0° and 135°), the same rms value of the residual current is forced. It is not difficult to notice that the delay angle equal to 135° gives a significantly higher peak value of the induced voltage than the case with a delay angle of 0°. For this reason, the tripping threshold of the RCDs, which depends on the peak value, is expected to be relatively low (for the angle of 90° or 135°), even below 0.5*I*_Δn_. 

## 4. Conclusions

Residual current devices are commonly used in modern low-voltage electrical installations. Their application in circuits of increased electric shock risk is even obligatory. However, the wide-spread application of current-using equipment supplied via power electronics converters makes proper operation of the most popular RCDs—A-type RCDs—doubtful. The most problematic RCD operation is expected in circuits utilizing high-frequency PWM modulation. The earth fault current comprises high-order harmonics, and the tripping current of the RCD can be very high; it can be many times higher than its rated residual operating current. For one of the tested RCDs (RCD30_4), having a rated residual operating current *I*_Δn_ = 30 mA, the tripping threshold was 320 mA (Figure 14). Even worse, some RCDs may not react to the non-sinusoidal current of a high content of high-order harmonics, as it has been shown in the above-presented investigations that no reaction was obtained in case of RCD30_2. In such circuits, protection against electric shock may not be effective. On the other hand, strongly distorted earth fault current, characterized by symmetrical delay angle, causes an opposite reaction of the RCDs; the tripping threshold can be clearly lower than for the sinusoidal waveform. During the laboratory test of the example RCD of *I*_Δn_ = 300 mA (RCD300_2), its tripping current was equal to around 100 mA (delay angle 135°), whereas for the sinusoidal waveform, it was around 220 mA. Thus, evaluation of the protection against electric shock in the presence of harmonics, if RCDs are to be used, should be performed in detail, including their additional laboratory testing.

The conducted research also gives key information for the modification of the structure of voltage-independent RCDs, dedicated to the circuits characterized by earth fault current with harmonics, especially high-order harmonics. As the sensitivity of RCDs is mainly determined by the frequency response of both the current sensor and the electromechanical relay, the material used for construction of their magnetic path has to have favorable properties for higher frequencies (narrow hysteresis loop, low level of losses). Moreover, the coil of the relay should be suitable for higher frequencies—the lowest number of turns (as much as possible) is recommended to be applied. It gives a relatively low level of reactance that depends on the frequency. Special attention must be given to the selection of the additional components (e.g., capacitors) installed in the secondary circuit of the current sensor. Capacitors may give a positive effect for the nominal frequency (50 Hz), but for other frequencies, the effect may be quite opposite. The behavior of the secondary circuit, including the additional components, has to be verified within the frequency range to which the RCD is dedicated. After such verification, the permissible range of the operational frequency should be published by manufacturers of RCDs.
